# Nutrition, Microbiota and Role of Gut-Brain Axis in Subjects with Phenylketonuria (PKU): A Review

**DOI:** 10.3390/nu12113319

**Published:** 2020-10-29

**Authors:** Elvira Verduci, Maria Teresa Carbone, Elisa Borghi, Emerenziana Ottaviano, Alberto Burlina, Giacomo Biasucci

**Affiliations:** 1 Department of Paediatrics, Vittore Buzzi Children’s Hospital-University of Milan, Via Lodovico Castelvetro, 32, 20154 Milan, Italy; 2 Department of Health Science, University of Milan, via di Rudinì 8, 20142 Milan, Italy; elisa.borghi@unimi.it (E.B.); emerenziana.ottaviano@unimi.it (E.O.); 3 UOS Metabolic and Rare Diseases, AORN Santobono, Via Mario Fiore 6, 80122 Naples, Italy; mt.carbone@santobonopausilipon.it; 4 Division of Inborn Metabolic Diseases, Department of Diagnostic Services, University Hospital of Padua, Via Orus 2B, 35129 Padua, Italy; alberto.burlina@unipd.it; 5 Department of Paediatrics & Neonatology, Guglielmo da Saliceto Hospital, Via Taverna Giuseppe, 49, 29121 Piacenza, Italy; g.biasucci@ausl.pc.it

**Keywords:** diet, nutrients, microbial, inherited errors of metabolism, immune response, neurological function, anxiety, depression

## Abstract

The composition and functioning of the gut microbiota, the complex population of microorganisms residing in the intestine, is strongly affected by endogenous and exogenous factors, among which diet is key. Important perturbations of the microbiota have been observed to contribute to disease risk, as in the case of neurological disorders, inflammatory bowel disease, obesity, diabetes, cardiovascular disease, among others. Although mechanisms are not fully clarified, nutrients interacting with the microbiota are thought to affect host metabolism, immune response or disrupt the protective functions of the intestinal barrier. Similarly, key intermediaries, whose presence may be strongly influenced by dietary habits, sustain the communication along the gut-brain-axis, influencing brain functions in the same way as the brain influences gut activity. Due to the role of diet in the modulation of the microbiota, its composition is of high interest in inherited errors of metabolism (IEMs) and may reveal an appealing therapeutic target. In IEMs, for example in phenylketonuria (PKU), since part of the therapeutic intervention is based on chronic or life-long tailored dietetic regimens, important variations of the microbial diversity or relative abundance have been observed. A holistic approach, including a healthy composition of the microbiota, is recommended to modulate host metabolism and affected neurological functions.

## 1. Introduction

Gut microbiota is defined as the complex population of microorganisms residing in the intestine with the highest concentrations being found in the colon [1]. In the last decade, the importance of the microbiota has been extensively studied, with particular attention to the implications it has on animal and human health. Diet has been identified as a major factor influencing gut microbiota composition and function, and all the diseases that require a dietary intervention as part of their therapeutic approach represent a target for both the research and treatment options.

A summary of the influence of food constituents as well as different dietary approaches may have on the microbiota follows.

### 1.1. What Influences the Microbiota

The gut microbiota features hallmark characteristics throughout the different life phases, starting with a relatively simple microbial composition to mature with an increased microbiota complexity associated to genetic as well as environmental and physiological influences, such as nutrition, lifestyle, hormonal changes, immunity, and the microbiome-gut-brain axis crosstalk. In humans, microbial communities also seem to vary substantially due to geographic location [2]. The trillions of microorganisms constituting the gut microbiota include more than a hundred bacterial species, divided into six phyla: Firmicutes, Bacteroidetes, Proteobacteria, Actinobacteria, Verrucomicrobia and Fusobacteria. Despite interindividual differences, the Firmicutes (such as *Clostridium*, *Enterococcus*, *Lactobacillus*, and *Ruminococcus* genera) represent up to 60% of the gut microbiota, the Bacteroidetes (such as *Bacteroides* and *Prevotella*) represent up to 15%, with all other species represented in a smaller proportion [3,4,5].

Among the most studied microbial genera in the human gut, the genus *Prevotella* has been shown to be mostly anti-inflammatory and protective [6], the genus *Bacteroides* to have pro-inflammatory effects and more often associated to increased risk of metabolic syndrome and other diseases, and the genus *Ruminococcus* to be further explored as its role in human health is less defined [7].

A factor to take into account is the role of some xenobiotics able to trigger gut microbiome toxicity. Among the most well-known are antibiotics, with numerous non-antibiotic drugs being tested recently too [8]. Short-term and long-term antibiotic treatments have an impact on the composition of the microbiota, determining diversity reduction and also changing the microbiota functional profiles [9]. Less studied is the role of gut bacteria as important bio-transformers of heavy metals, able to promote or attenuate toxicity of xenobiotics. Exposure to heavy metals has been shown to modify the composition and the functionality of the microbiota in rats [10]. 

Of interest is the case of artificial sweeteners, food products generally considered safe because they are poorly metabolized by the human body. The microbiota, though, are very active in the process of biotransformation and some of these products may be actively metabolized and give rise to toxic species [11,12,13]. Furthermore, other studies demonstrated that some artificial sweeteners are able to induce gut microbiota alterations that can contribute to the development of health issues. Saccharin involved in the development of glucose intolerance [14] or associated to increased inflammatory levels and gut microbial perturbation [15] reflect this circumstance in pre-clinical models. Other artificial sweeteners, namely acesulfame potassium [16], sucralose [17], aspartame [18], and neotame [19], have been found to perturb bacterial metabolite production in concert with health implications including obesity and inflammation.

Although it is difficult to disentangle the complexity of the relationship of microbiota with the human body and its metabolism, changes in microbial metabolites, diversity loss, and interference in energy metabolism could be considered among the most important disturbances that may impact host health via multiple host–microbiota axes. Not all the disturbances are necessarily negative; but if they are, they can potentially lead to increased disease risks [9].

### 1.2. How Diet Modulates the Microbiota

More data sustain the hypothesis that environmentally-induced perturbations are potentially linked to elevated disease risks [20,21]. Adverse health outcomes including inflammatory bowel disease, obesity, diabetes, cardiovascular disease, liver disease, colorectal cancer, and neurological disorders can be at least in part sustained by undesirable functional alterations in the gut microbiome [22,23,24,25,26,27,28]. A good number of studies in humans [29,30] have shown that diet-induced alterations of gut microbiome can occur rapidly and in a reproducible manner. For example, within four days of an entirely animal-based or plant-based diet, the microbiome results altered [29]. Furthermore, it has been shown how, differently from the simpler and more homogeneous diets in rural areas, urban environments offer a large variety of foods, leading to greater inter-individual variability of gut microbiota [31].

The way nutrients interact with the microbiota can be direct, by providing, or not, the necessary dietary constituents for the growth of certain microorganisms over others, or indirect by affecting host metabolism and immune response or by disrupting protective functions of the intestinal barrier, for example, prompting dysbiosis, and so contributing to inflammatory processes [32].

Looking into the quality and composition of the diet, metagenomic and metabolomic analysis studies have shown differences in the microbiota composition as a result of a trade-off between protein fermentation and degradation in protein-rich diets as opposed to carbohydrate fermentation and AA biosynthesis in plant-based diets [29,33]. Furthermore, microbiome gene richness has been reported to be positively correlated with the consumption of fruits, vegetables, and fish in overweight or obese humans [34].

Preclinically, in mice fed a high-fat or a high-fat/high-sugar ”Western” diet, a decrease in Bacteroidetes levels and an increase in Firmicutes and Proteobacteria has been shown to be dose-dependent [35,36,37]. The change in the composition of the microbiota of mice fed the “Western diet” was accompanied by a functional change, with increased sucrose and urea metabolism, modification in the membrane transport systems, or metabolism of cofactors [38,39].

Modifications in the relative abundance of genera in the human gut may be associated with perturbations correlated to the specific effects of the bacteria metabolism. For example, *Ruminococcus gnavus* has been associated to a pro-inflammatory effect in light of the production of a pro-inflammatory polysaccharide in patients with Chron’s disease [40]. Bacteria of the genus *Clostridia* of the Firmicutes phylum have been thoroughly studied as they produce several toxic substances such as phenol, indole, and p-cresol able to reduce the growth of lactic acid bacteria [41]. Among *Clostridia*, *Clostridium difficile* is well characterised as it produces toxins A and B, which in turn induce potent pro-inflammatory signalling in the human body [42]. On the contrary, the butyrate-producing *Faecalibacterium prausnitzii* is an example of a marker of gut health in light of immunosuppressive features of the short-chain fatty acids (SCFA) it produces [43].

Furthermore, the relative abundance of microbial species is of interest. For example, the balance between Bacteroidetes and Firmicutes is an important marker for obesity and higher BMI. A decreased Bacteroidetes:Firmicutes ratio has a strong negative correlation with BMI [44], possibly related to the observation that a 20% increase in Firmicutes and a corresponding decrease in Bacteroidetes abundance is associated with a 150 kcal/day increase in energy harvest, resulting in weight gain overtime. Therefore, an increased Bacteroidetes:Firmicutes ratio, as seen on a high fibre, plant-based diet, may result in weight loss by reducing the amount of energy extracted from the diet, but further data are needed to exactly understand the underlying mechanism of action [45].

Despite interesting research results, in man, the multitude of variables interfering with their lives can have synergistic or opposing outcomes on the gut microbiome, making it difficult to anticipate the net effect of dietary interventions on the microbiota downstream on the human host. Nevertheless, deficiencies of some micronutrients have been found to trigger distinct patterns of microbiota structural alterations in humans, mice, rats, and piglets. Noteworthy examples include iron [46,47,48,49], magnesium [50], zinc [51,52], selenium [53], nitrite or nitrate [54], vitamin A [55], and vitamin D [56].

The research is always providing more data clarifying the role of nutrition in modulating the microenvironment, but a key factor to always be taken into account remains the inter-individual variability, determined by person-specific responses, [57] and depending not only on microbiota composition but also on host metabolism, meal timing, nutritional content, and exercise [31].

A summary is reported in Table 1 indicating how macronutrients, micronutrients, and food additives interact with the microbiome. This summary also highlights how the relative abundance of specific genera, within the complex network of interactions, may bring both beneficial and detrimental effects on the host [32].

### 1.3. Role of Carbohydrates, Lipids, Proteins and Amino Acids on Microbiota

As this review is focused on patients with phenylketonuria (PKU) who need to heavily modify their dietary regimens in order to reduce intake of phenylalanine (Phe), it seems of value to evaluate the respective roles of proteins, carbohydrates, and lipids in the modulation of the microbiota. Patients with PKU receive a long, ideally life-long, low-protein diet, compensated by the regular supplementation of Phe-free amino acids (AAs), and may receive an excess of low-protein carbohydrates and lipids.

#### 1.3.1. Carbohydrates

Gut microbiota can be modified by dietary carbohydrates, and carbohydrates have a role in the bacterial metabolism. Depending on the type of diet administered, the gut microbiota is modified with bacteria species resulting more or less abundantly as a response to the dietary regimen. Similarly, complex polysaccharides or non-digestible carbohydrates (resistant starch, non-digestible polysaccharides, oligosaccharides, and plant fibres) can be processed by the gut microbiota supporting their digestion or using indigestible carbohydrates as a source of energy for their own metabolism [69,70].

SCFAs, small organic monocarboxylic acids with a chain length of up to six carbons atoms, such as lactate, acetate, propionate, and butyrate, produced by the microbiota via carbohydrate fermentation, may have beneficial effects, positively modulating carbohydrate and lipid metabolism, energy homeostasis, intestinal barrier function, mucosal immune homeostasis, and inflammatory signal inhibition in the host [58,59,63]. 

However, their effects have also been found to contribute to enhanced energy harvest in obese humans and ob/ob genetically obese mice, suggesting unwanted implications in obesity status [71]. Data obtained in ob/ob mice confirmed this observation, indicating a 50% reduction in the abundance of Bacteroidetes and a proportional increase of Firmicutes, a factor that might contribute to promote adiposity [45]. In humans, several studies have shown that the ratio of Firmicutes to Bacteroidetes significantly affects body mass index (BMI) and childhood obesity [72].

#### 1.3.2. Lipids

The ratio and source of dietary fat are equally important to intestinal microbiota modifications [73]. The consequences of plant- and animal-based fat sources on gut microbiota are notably different, with plant-based fats observed to increase *Bifidobacterium*, *Roseburia*, and *Faecalibacterium*, associated with positive metabolic effects [74]. Analogously, saturated and unsaturated fats have been shown to modulate the microbiota differently. Highly saturated fat diets have been associated with an increased ratio of gram-negative intestinal microbial species and increased endotoxemia [68] or increased intestinal permeability [67]. While gut microbiota can be modified by dietary fats, microbes may modulate lipid metabolic abilities of the host by regulating absorption, storage, and energy harvest from the diet [75,76]. Among the numerous studies, an interesting one, in a population of 893 subjects from the Life-Lines-DEEP population cohort, identified 34 intestinal bacterial taxa correlating with BMI and blood lipids. They found gut microbiota to be responsible for 4.5% variation in BMI, 6.0% in triglycerides, and 4% in high-density lipoproteins (HDL), independently of age, sex, and genetic risk factors [77].

Another exploratory experience, a placebo-controlled, randomized, double-blind study showed that six months (but not three months) of *Lactobacilli* and *Bifidobacteria* supplementation (50 billion cfu/day) significantly reduced bodyweight, BMI, waist circumference, and waist-to-height ratio in free-living overweight/obese subjects, with females benefiting more than male participants. Greatest weight losses together with decreases in small dense LDL-C levels were observed in hypercholesterolemic participants [78].

#### 1.3.3. Amino Acids

Literature reports that some metabolites of AAs such as ammonia, amines, phenol and p-cresol are detrimental for the colonic mucosa at high concentrations [79,80,81] but also that protein-derived metabolites i.e., amines and indole, may play important roles in the regulation of normal intestinal functions at physiological concentrations [81,82]. Indole can enhance the intestinal barrier function and attenuate inflammation [83], while polyamines seem to exert protective effects on intestinal mucosa [84,85].

Different studies have highlighted the role of single AAs on gut microbiota and consequently on intestine performance or health. Glutamate, glutamine, and aspartate are the major oxidative fuels of the intestine. New evidence shows that arginine activates the mTOR signalling pathway in the small intestine, while glycine, used to synthesize glutathione in the small intestine, is considered a powerful cytoprotectant. Furthermore, the major end products of methionine and cysteine metabolism, glutathione, homocysteine, and taurine, play important roles in the intestinal immune and anti-oxidative responses. Threonine is highly utilized by the gut and is particularly important for mucin synthesis and maintenance of gut barrier integrity [64]. Of interest, data seem to suggest that in obesity and associated metabolic disorders, gut microbiota dysbiosis likely plays a significant role in decreasing the availability of glycine, an important AA required for multiple metabolic pathways [65].

Based on the observation that in pigs a moderate reduction of dietary protein levels (≤4%), compensated by the supplementation of essential AAs, reduces the proportions of harmful bacteria and protein fermentation without damaging the growth performance of pigs, Wang H. et al. compared the effects of casein hydrolysate and free AA supplementations together with a low-protein diet. Their data show that in the casein hydrolysate group the abundance of potentially pathogenic bacteria was reduced while *Lactobacillus* and short-chain fatty acid concentration, expression of T-helper type 2 cytokines and Mucin-4 increased when compared with free AA supplementation [86]. 

Another pre-clinical study in a streptozotocin-induced type 2 diabetes mouse model suggests that the addition of glycomacropeptide (GMP) hydrolysate to a high-fat diet contributes to an anti-diabetic effect by recovering the muscle insulin sensitivity and by modulating the microbiome. The latter has been found to present an increased ratio of Bacteroidetes/Firmicutes, suggesting that GMP may exert its antidiabetic effects via regulating the integral structure of gut microbiota [87].

A very recent study in newborn rhesus monkeys showed the role of adding free AAs in formulas of new-borns whom, differently from those who were breast-fed, are known to exhibit more rapid weight gain, a different faecal microbial profile, as well as elevated serum insulin, insulin growth factor 1, and branched chain AAs (BCAAs) levels. The removal of proteins and the addition of free AAs, in an effort to provide food more similar to breast milk, profoundly suppressed formula intake, lowered weight gain, and improved serum insulin concentrations. Levels of faecal microbes known for their ability to utilize complex carbohydrates (*Bifidobacterium* and *Ruminococcus* from the *Ruminococcaceae* family), increased with protein reduction, although the overall microbial composition did not significantly change. While the addition of free AAs allowed observing of growth rates and metabolic performance of formula-fed infants more similarly to those of breast-fed infants, it resulted in being insufficient to reverse the accelerated growth and the insulin-inducing high BCAA phenotype, typically observed in formula-fed monkey new-borns [88].

## 2. The Gut-Brain Axis

**Figure 1:**
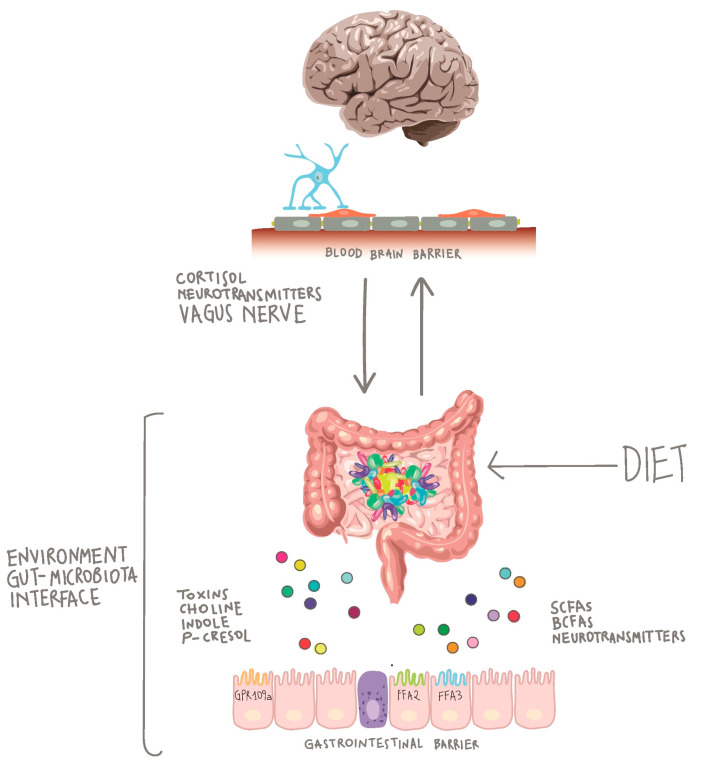
Microbial role in the bidirectional communication between brain and gut. SCFAs: short-chain fatty acids; BCFAs: branched-chain fatty acids.

Another fascinating topic regarding the crosstalk between the gut microbiota and the central nervous system (CNS), is in its infancy as regards the understanding of the involved mechanisms and the number of clinical studies. The gut microbiota can communicate with the CNS by means of five different communication routes: the neuroanatomical pathway, the neuroendocrine-hypothalamic–pituitary–adrenal axis pathway, the immune system, the gut microbiota metabolism pathway, and the intestinal mucosal barrier and blood-brain barrier [89]. The flow of communication can be bi-directional: “bottom-up” from the gut microbiota to the brain and “top-down” from the brain to the gut microbiota (Figure 1). The bottom-up communication mainly occurs through neuroendocrine and neuroimmune systems, involving the enteroendocrine cells and the gastrointestinal enterochromaffin cells, the intestinal mucosal barrier, and the blood-brain-barrier. The metabolites of the gut microbiota, such as SCFAs, tryptophan (Trp) metabolites, and secondary bile acids, are key players mediating this bottom-up communication [90,91]. On the other hand, the top-down communication between the brain and gut microbiota involves the neuroanatomical pathway, the regulation of the intestinal barrier, and the release of neurotransmitters (e.g., 5-HT and catecholamines) [92,93,94].

Immune cells (e.g., macrophages and dendritic cells) and enteric glial cells (EGCs) constantly sense microbial-associated molecules produced at gut level. In response to these stimuli generated by the microbial population, neuro-modulatory molecules targeting the enteric nerves can be secreted locally. Once the enteric nerves are stimulated, they can, in turn, affect the brain and the spinal cord by means of vagal transmission. When molecules or stimuli reach the CNS, neurons, microglia, and astrocytes respond by changing their transcriptional program, either supporting or inhibiting pathological conditions. Increasing knowledge is shedding light on the mechanisms underlying microbiome-gut-brain axis in different pathologies, including neuropsychiatric disorders or neurodegenerative and metabolic diseases [95,96,97].

At gastrointestinal level, the autonomic nervous system is known to regulate gut motility and permeability, secretion of bile acids, bicarbonates, and mucus, as well as mucosal immunity [98]. Moreover, the autonomic nervous system can influence the intestinal barrier integrity directly altering the permeability of intestinal epithelial cells and modulating the intestinal mucus layer [90]. It has been reported that some lactobacilli (*Lactobacillus reuteri* and *L. rhamnosus*) improve the enteric nervous system and vagus nerve activations [99,100] while others (*L farciminis*, *L. Plantarum*, and *L. fermentum*) can produce a neurotransmitter, nitric oxide, associated with control of the intestinal transit [101,102].

One of the most studied topics in gut microbiota modulation is the role of SCFAs, released by the microbiota in the intestine as the main products of the anaerobic fermentation of indigestible dietary fibres and resistant starch. SCFAs can be systemically distributed and cross the blood-brain-barrier where they seem to have numerous properties, including neuroactive functions. The exact mechanisms by which SCFAs exert their effects are largely unknown but several animal studies have shown an influence on key neurological and behavioural processes that may be involved in neurodevelopmental and neurodegenerative disorders [103,104,105,106,107].

Despite the complex pathophysiology of mood disorders, there are studies highlighting the participation of the gut microbiota in the severity of these diseases [108]. For example, the importance of the microbiota in depression is supported by findings that the levels of SCFAs are decreased in a naturally occurring non-human primate model of depression [109] and by clinical evidence showing that faecal SCFA concentrations are lower in patients with depression than in controls [110,111]. Furthermore, it has been shown that Bacteroides can produce γ-aminobutyric acid (GABA) [112], an inhibitory neurotransmitter that modulates brain function and emotional behaviour via the vagus nerve [113].

Intestinal microbiota represents an essential bioreactor that converts dietary substrates into metabolites with a crucial role in a variety of physiological processes. SCFAs are the most abundant products of microbial fermentation, but neurotransmitters (for example γ-aminobutyric acid and serotonin), choline, phenol derivatives, and toxins are also produced [114]. A bottom-up communication, mainly through microbial products engaging cellular receptors (fatty acid receptors) on the gastrointestinal epithelium [90,91], can modulate peripheral and central effects modifying host metabolism [115]. Vice versa, top-down regulation is mainly mediated by the vagus nerve, which can modulate the intestinal milieu by reducing intestinal mobility and permeability and exerts anti-inflammatory properties [92,93,94].

Therefore, the diet has a role, especially when taking dietary extremes (e.g. a Mediterranean diet vs. a Western-style diet) into account, in the way the gut-brain axis works. Key intermediaries, more or less also present based on the dietary habits, sustain the communication along the gut-brain axis. The microbiota, producing metabolites such as serotonin/5-hydroxytryptamine, GABA, glutamate, Phe, Trp, tyrosine (Tyr), carnosine, SCFAs, threonine, alanine, lysine, glycine, serine, aspartic acid, ammonia, and gut hormone/incretins, may influence brain functions as well as brain influences on gut activity. In addition, the gut microbiota has the ability to regulate the barrier integrity (both within the gut and at the blood brain barrier), and partial nutrient absorption at the mucosal surface [116].

## 3. Microbiota Modulation for Therapeutic Purposes

A great deal of attention has been placed on the potential contribution of the microbiota to disease pathogenesis and clinical manifestations, making it an appealing therapeutic target for treatment of selected diseases. For example, in case of metabolic syndrome, a group of co-associated diseases including obesity, insulin resistance, type 2 diabetes, cardiovascular disease, and non-alcoholic fatty liver disease, the research is currently focusing not only on the analysis of bacterial community composition and how they associate with the disease, but is also trying to elucidate the molecular pathways and metabolites activated and produced by the microbial community, to understand what their effects on manifestations related to metabolic syndrome could be [117]. The observation that the microbiota can benefit from some foods, even if indigestible for humans, has sustained the rise of prebiotics, non-digestible foods beneficial for selectively stimulating either the growth, activity, or both, of bacterial species resident in the gastrointestinal tract, with the consequence of improving human health [118].

Prebiotics may support the positive action of probiotics [119], the latter developed as commercially available viable microbial supplements. If appropriately combined as synbiotics [120], pre- and probiotics may even exert superior effects on enhancing health functions [121].

Although more data are necessary before they can be fully understood and successfully used, there seem to be fairly strong indications that this approach may be beneficial [117]. As an example of how widespread the use of these products could be, beneficial effects of probiotics in human health have been reported, among others, in neurodevelopmental [122,123] and neurological diseases [124,125,126,127,128], diabetes [129,130,131], obesity [132,133,134,135], and metabolic syndrome [136], and in inflammatory diseases [137,138] and allergies [139,140,141].

**Figure 2:**
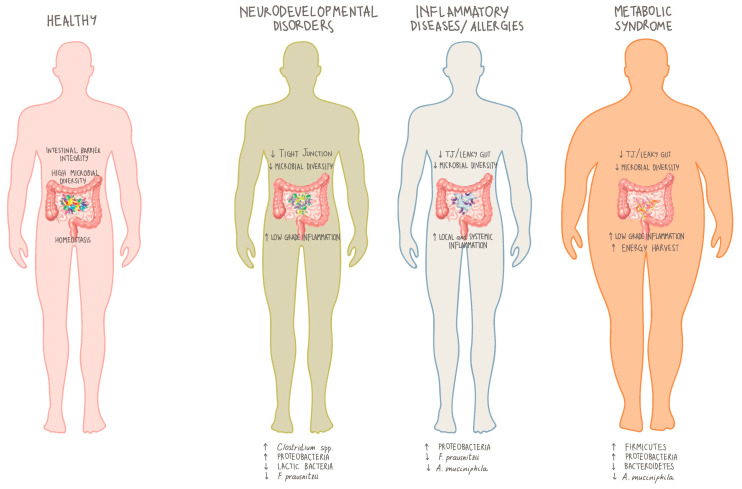
Microbial signature in health and disease and its impact on gastrointestinal homeostasis. Up and down arrows indicate increase and decrease, respectively. TJ = tight junction.

Loss of microbial diversity represents the first step toward an altered microbial environment, promoting host susceptibility to diseases (Figure 2). Indeed, it is a common feature in the reported pathological conditions: neurodevelopmental disorders [122,123], inflammatory diseases [137,138], allergies [139,140,141], and metabolic syndrome [136]. Similarly, in all conditions, an increase in proinflammatory taxa (i.e., Proteobacteria) is reported, suggesting microbial participation in local and systemic low-grade inflammation [142,143,144].

Green et al. [144] very recently published a review on the role of pre and probiotics in obesity and metabolic syndrome. The microbiome is involved in the generation of disease states, although the interplay of the many factors involved is not fully understood. The wealth of available literature strongly implicates that gut dysbiosis as a key contributor to obesity development and associated metabolic abnormalities, and modulation of the microbiota with pre- and probiotic foods represent an avenue for potential therapeutic interventions. For example, incorporating either fermentable carbohydrates, strains of *Lactobacillus*, *Bifidobacterium*, and other select taxa, or both, into the diet not only mediates improvements to body weight and adiposity but exerts other positive effects on glycaemic control, systemic inflammation, and energy intake. It is necessary, though, to carefully study how these therapeutic approaches work in non-healthy populations, as indicated by some studies suggesting that certain bacterial species of these same genera may be ineffective if not deleterious for obese patients [144].

Among the microbiota roles, the ability to regulate the functionality and maturation of the host’s neuroimmune system [145,146] is key for the development and the functioning of the brain. In fact, it has become more and more clear that a healthy gut microbiota is required for unaffected brain development in the postnatal period and infancy years as well as for cognition in adulthood [147,148,149,150]. Gut microbiota has also been correlated to the aetiology of neuropsychiatric disorders, including anxiety [151] and depression [107,152]. In addition, chronic stress has been shown to favour leaky gut syndrome related to low-grade inflammation that can be functionally related to neuropsychiatric disorders [153].

It therefore remains important to continue studying specific patient populations, especially in presence of diseases such as the inherited errors of metabolism (IEMs), where often a modified dietary regimen needs to be followed for many years, if not life-long, with numerous consequences. 

Initial assessments of these disorders have been conducted, and discussed below, helping to increase the know-how that will ultimately allow clinicians to personalise the use of pre and probiotics to the benefit of the patients.

## 4. Microbiota and Inherited Errors of Metabolism (IEMs)

As concerns IEMs, the last few years have seen increasing research data being published on the influence of the dietary regimen on microbiota, due to the fact that at least a part of the therapeutic intervention in patients with these disorders occurs by means of specifically tailored dietetic regimens.

These diseases, which share the absent or deficient activity of a given enzyme, show high genetic ad epigenetic heterogeneity and also show different responses among patients with the same genotype. In untreated phenylketonuria (PKU) and in propionic and methylmalonic acidaemia, for instance, the neurological and behavioural impairment are highly variable, while liver disease is commonly developed in tyrosinemia type 1 and urea cycle disorders [154]. Despite such heterogeneity, treatment approaches generally include one of the following: (I) enzyme replacement therapy, to replenish the deficient enzyme; (II) substrate reduction therapy; or (III) dietary treatment (or organ transplantation in selected cases) [155]. The purpose of the dietary treatment in IEMs is to reduce the target toxic compound that accumulates in the body [156], but contemporarily it may cause either dangerous overloads, deficiencies of certain food groups and nutrients, or both [157,158]. Examples are diets that result in restricted or are excessively rich in certain nutrients and that may prompt intestinal dysbiosis with systemic effects such as malnutrition, obesity [159], type 1 [160] or type 2 diabetes [161], inflammatory bowel disease [162,163], liver disease [164], symptoms of autism spectrum disorders [165], and even cancer [166,167].

Another aspect that needs to be taken into account in IEMs is the susceptibility of the central nervous system and the liver due to their high metabolic rate [168]. A few publications indicate how the microbiota may influence the CNS and consequently exert unwanted effects on metabolism [77,169], coordination [170], mood [171], behaviour [172], cognition [173], temperature control [174], and sensation [175].

Considering the high diversity of IEMs and the relative scarcity of patients in each type of condition, literature on the role of microbiota in these diseases is infrequent for the rarest types. In aminoacidopathies, the majority of the data are available in PKU, while limited data have been published in tyrosinemia and alkaptonuria [176], classical homocystinuria [177], and organic acidaemias (methylmalonic acidaemia and propionic acidaemia) [178,179]. Nevertheless, the common trait of the available data indicate that, based on the specific intervention (either diet, drugs, or both), the microbiome can be positively or negatively influenced, underlining how the elucidation of the metabolic role of the single species constituting the microbiota—especially if influenced by treatments as in the case of IEMs—may allow appropriate manipulation in the direction of stimulating pathways beneficial to the patient and inhibiting harmful ones.

In type I nitisinone-treated tyrosinemia patients, a perturbation of the indole metabolism associated with high levels of 4-hydroxyphenylpyruvate has been shown to be directly responsible for the promotion of the indole-pyruvate pathway via the Trp metabolism. Despite the fact that the influence of indolic compounds on human cellular function has not been well described, this class of compounds is especially important in bacterial function and signalling, making perturbations of this pathway potentially interfering with the human microbiome, and consequently the patient [176].

Classical homocystinuria (HCU) is characterized by increased plasma levels of total homocysteine and methionine and part of its treatment consists of supplementation of B vitamins, essential AAs, and a dietary regimen aimed at restricting methionine intake. As dysbiosis has been described in other inborn errors of metabolism, interest in this topic promoted conduction of clinical study in HCU patients on dietary treatment. Among other results, this study indicated that patients with HCU had overrepresentation of the *Eubacterium coprostanoligenes* group and underrepresentation of the *Alistipes*, Family XIII UCG-001, and *Parabacteroides* genera, which have been associated, among other factors, to the diet and vitamin B supplementation [177].

In propionic and methylmalonic acidaemias, the gut microbiota significantly contributes to the production of propionic acid [180,181] a metabolite that together with methylmalonic acid is accumulated due to enzyme malfunctioning. Because of the participation of the microbiota to the production of propionate, potentially exacerbating the symptomatology in these patients, the microbiota itself may be considered a therapeutic target. In a study on lymphoblastoid cell lines from patients with autism spectrum disorder, mitochondrial function was measured in presence of propionic acid in increasing concentrations and durations and in presence or absence of reactive oxygen species (ROS). Mitochondrial function was optimally increased at particular exposure durations and concentrations of propionic acid in cell lines deriving from patients with autism spectrum disorder. Conversely, increasing the presence of ROS negated the positive effect of propionic acid in these cell lines. The enteric metabolites produced by the microbiota, of which propionic acid is an example, can have both beneficial and toxic effects on mitochondrial function, depending on concentration, exposure duration, and microenvironment redox state [178]. Based on the fact that the modulation of the diet and the potentially different microbiota composition due to the disease can influence propionate production, as well as the capacity of pre or probiotics to produce propionate, it has been hypothesized that a potential approach aimed at reducing propionate production, in combination with currently indicated interventions, could be the administration of prebiotics such as galacto-oligosaccharides and fructo-oligosaccharides. These prebiotics are able to significantly increase acetate and lactate while reducing propionate and butyrate, although their role in clinical practice needs first to be confirmed in clinical studies assessing the extent of the reduction of propionate production in the gut, and possibly also how the modulation of the dietary regimens may contribute [179].

Another example of interference of microbiota with metabolism is represented by carnitine, widely supplemented to patients with organic acidaemias and primary carnitine uptake deficiency [182]. Because carnitine is a precursor of trimethylamine N-oxide (TMAO), and the latter has been identified in a dose dependent way as a risk factor for cardiovascular disease, potentially advancing atherosclerosis [24,183,184], patients with IEMs receiving L-carnitine may be at risk. Restriction of meat in the dietary regimen attenuates conversion of carnitine to TMAO, but due to the role of intestinal microbes in the TMAO conversion, metabolomic analyses have been carried out in patients with IEMs supplemented with L-carnitine. Marked plasma TMAO elevations were detected in patients treated with supplemental L-carnitine, including those on a meat-free diet, elevated TMAO levels up to ~45-fold in patients with organic acidaemias as compared with the reference population. The current approach of administering a 7-day therapy with metronidazole to reduce TMAO levels could be accompanied by strategies to circumvent intestinal bacteria to improve L-carnitine therapy [182].

Similarly, in glycogen-storage diseases (GSD-Ia and Ib), a recent publication reported the results of a study, where the gut microbiota of 9 GSD-I subjects and 12 healthy controls was compared. The analyses revealed a significant biodiversity reduction in the GSD group compared to the healthy control group highlighting profound differences of the respective gut microbiota. GSD subjects were characterized by an increase in the relative abundance of Enterobacteriaceae and *Veillonellaceae* families, while the beneficial genera *Faecalibacterium* and *Oscillospira* were significantly reduced. SCFA quantification revealed a significant increase of faecal acetate and propionate in GSD subjects, whose beneficial role was probably reduced due to unbalanced bacterial interactions [185].

## 5. Role of Microbiota in PKU

### 5.1. Article Search

We performed a search of the literature in the PubMed and Google Scholar databases to identify articles related to “phenylketonuria” and “microbiota”/“microbiome”, executed on 10 May 2020. Due to the scarcity of literature published, all articles were included in this review with the exception of duplicates. A total of 13 eligible articles (6 preclinical and 7 clinical articles) were identified and reported.

### 5.2. Preclinical Studies

So far, the role of microbiota has been studied with more depth in PKU than in any other IEM. A handful of pre-clinical works have been published both to shed light on mechanisms involving the microbiota in PKU animal models and to describe how approaches to the microbiota my benefit patients with PKU.

The first study to start analysing how microbiota may be modulated in PKU was published in 2015 by Sawin E.A. et al. [186], to describe how glycomacropeptide (GMP), a highly glycosylated natural protein source composed of a 64-amino acid peptide derived from cheese manufacturing process and nowadays among the protein substitute options for patients with PKU, showed prebiotic features. Based on the observation that GMP increased *Lactobacillus* and *Bifidobacterium* populations in mice in as little as three days after treatment, the study, conducted in PKU (*Pah^enu2^*) and wild-type mice, evaluated GMP as a prebiotic by characterizing caecal and faecal microbiota populations, SCFA production and immune responses. Considering that changes in caecal and faecal microbiota are primarily diet-dependent, the introduction of GMP into the diet was associated with a reduction in *Proteobacteria*, genera *Desulfovibrio*, in both wild-type and PKU mice. The genus *Desulfovibrio*, associated with production of hydrogen sulphate, is a cytotoxic compound found at higher levels in patients with ulcerative colitis [187]. Furthermore, caecal concentrations of SCFAs, acetate, propionate, and butyrate increased in mice receiving GMP, contrary to biomarkers of inflammation (IFN-g, TNF-a, IL-1b, and IL-2), found reduced in mice fed GMP without affecting circulating Phe levels [186].

More recently, another pre-clinical study investigated if microbiota could influence tetrahydrobiopterin (BH_4_) presence, due to the existence of BH_4_-producing bacteria. GTP cyclohydrolase 1-deficient hyperphenylalaninaemia-1 (hph-1) mice were compared to wild-type controls for the presence of intestinal BH_4_-producing bacteria measuring 6-pyruvoyltetrahydropterin synthase (PTPS-2), an enzyme only present in BH_4_-generating bacteria. Adult wild-type mice and all-age hph-1 mice presented faecal material containing PTPS-2 mRNA, an indication of the presence of BH_4_-generating bacteria. In cultured human faecal samples, the PTPS-2-producing bacteria were identified as belonging to the Actinobacteria phylum. In hph-1 mice, *Aldercreutzia equolifaciens* and *Microbacterium schleiferi* were associated with bacterial production of BH_4_ [188].

Another study analysed the influence on the microbiota of GMP-based products when used in the artificial colon model, growing faecal material from healthy and frail older (non-PKU) subjects. The commercially available GMP-based product shown to sustain the growth of *Coprococcus* and *Clostridium* cluster XIVb and was associated with a higher faecal microbiota diversity compared to a control substrate (semi-purified GMP concentrate) or lactose. Differently, lactose fermentation promoted Proteobacteria growth, indicating that, depending on the substrate, influences on the relative abundance of gut microbial species may occur and in particular that the commercially available GMP was positively associated with the growth of health-related taxa in healthy subjects [189].

Other approaches consider the possibility to provide the gut with genetically engineered microbes able to degrade some of the ingested Phe prior to absorption, acting as a probiotic. The gene coding for the enzyme Phe lyase from *Anabaena variabilis*, a filamentous cyanobacterium, has been cloned into a vector to be expressed by *Lactobacillus reuteri*. Following in vitro confirmation that the *Lactobacillus* expressed a functional enzyme, the genetically modified bacteria have been tested in vivo in homozygous PKU mice. Reduced blood Phe levels were observed already after 3 to 4 days of treatment with the probiotic, and in other 2 experiments showed to maintain the Phe-lowering effect after 7 or 14 days of continued probiotic administration [190].

With an analogous aim, *Escherichia coli* Nissle has been engineered to express gene encoding Phe-metabolizing enzymes once in the mammalian gut, utilizing different metabolic pathways that produce harmless compounds including trans-cinnamic acid, further metabolized in the liver and excreted as hippurate in the urine. Tests have been conducted in Pah^enu2/enu2^ PKU mice to observe that the engineered bacteria could be associated with a reduced blood Phe concentration of 38% compared with wild type controls, irrespective of protein intake. The engineered *E. coli* has also been tested in healthy monkeys, where it resulted in being able to counteract the physiological Phe increase, following an oral Phe dietary challenge [191].

The murine model of PKU (Pah^enu2-/enu2-)^ has been used to assess if *E. coli* Nissle expressing PAL2 (Phe lyase from *Arabidopsis thaliana*) could significantly reduce serum Phe concentrations 24 h after probiotic gavage. The study revealed up to 50% reduction in serum Phe levels, but this was dependant on strain design and extent of hyperphenylalaninaemia [192].

### 5.3. Clinical Studies

Most studies on microbiota–IEM interactions have been focused on PKU.

One of the first attempts was carried out by MacDonald et al. [193], focusing on the potential impact of prebiotic treatment in PKU. The study aimed at analysing the effects of prebiotic oligosaccharides (scGOS/lcFOS) as an adjunct to the metabolic formula, the mainstay of management in infants with PKU. Being breastfeeding-restricted subjects, the authors theorized that a lack of the oligosaccharides normally present in breast milk might be associated with increased faecal pH and reduced bifidobacterial populations, thus predisposing the patient to infections. Postulating that administration of probiotics might show a mitigating effect, the study not only identified the dominant bacterial groups, but also showed that the prebiotic oligosaccharides could maintain bifidobacteria levels and low faecal pH, without altering circulating levels of Phe. As a first effort in a rare disease setting, despite the small sample size and lack of statistical power, this study suggests that supplementing metabolic formula with prebiotics might be an interesting strategy in PKU. At the end of the observation period, bifidobacteria and lactobacilli-enterococci were similar to those found in healthy children and higher than those reported in children who took the formula without prebiotics [193].

One of the first studies to assess the relative abundance of microbial species in the gut of very young patients (mean age: 4.24 years) with PKU, showed a reduced abundance of certain bacteria (*Clostridiaceae*, *Erysipelotrichaceae*, and *Lachnospiraceae* families, *Clostridiales* class, and *Coprococcus*, *Dorea*, *Lachnospira*, *Odoribacter*, *Ruminococcus*, and *Veillonella* genera) or an increase of others (*Prevotella*, *Akkermansia*, and *Peptostreptococcaceae* populations) when compared to that of healthy individuals. The study results suggest that in patients as well as in matched controls, the differences in the microbiota composition between patients and their matched controls may be ascribed to different dietary habits, such as the percentage of calories deriving from carbohydrates and lipids, the percentage of proteins and AAs coming from products of high biological value, and the intake of selenium, in addition to different Phe circulating levels. The authors suggest that key influencers of a different microbial species abundance in the guts of patients with PKU and healthy subjects could be due to either the disorder, in light of the different plasma Phe levels that appear to have contributed significantly to shifts in the gut microbiota, to the modified dietary habits needed to control Phe blood levels in patients with PKU [194], or both. Predictive microbial gene analysis showed an increase in genes related to lipopolysaccharide biosynthesis in patients with PKU that might be associated with peripheral inflammation, as suggested by the proinflammatory circulating cytokine profile of these patients [195]. Besides the differences in circulating Phe due to daily intake of AAs as well as to the different dietary regimens of the two groups, the higher blood Phe levels in patients with PKU have been supposed to have a role in the shift of the gut microbiota. Further studies are warranted in groups that do not have confounding factors, such as the intake of ferrous sulphate, vitamins, and prednisolone by PKU patients in the week preceding the stool sample collection, like in this study.

In addition to studies in animal models, GMP has also been characterized in patients with PKU, assessing its features under different angles. Albeit, Phe amounts provided by the GMP-based products, containing a residual concentration of Phe associated with the manufacturing process, should be added to the daily allowance [196], especially in certain categories of PKU patients, GMP-based products are quite commonly used nowadays.

The role of GMP as a prebiotic has been reported by Ney D. et al. [197] in a study aimed at assessing if the monoamine metabolites, important precursors of monoamine neurotransmitters, are differently produced based on the type of protein substitutes administered to patients with PKU. Metabolomic analyses revealed that microbiome-derived potentially harmful degradation compounds synthesized from Tyr and Trp were higher in patients receiving free AAs than those receiving GMP-based protein substitutes. The authors hypothesized that free AAs provide less bioavailable Tyr, partially due to a higher degree of Tyr metabolization by the resident gut bacteria, and also due to single smaller amounts of Tyr consumed by patients taking GMP-based products, who took protein substitutes more frequently over the day. There was no differential degradation of Trp, but the metabolism of Trp via the kynurenine pathway was evidenced by higher levels of metabolites linked to this pathway and might be linked with inflammation patterns [197].

Another study on GMP measured lipid metabolism in adult and adolescent patients with PKU on a low-Phe diet receiving free AAs or GMP-based products. The study was planned on the basis of the hypothesis that an altered neuronal lipid metabolism, promoting higher oxidation and inflammation rates, could negatively modulate cognition in PKU. Despite this study not directly measuring the effects of the different protein substitutes on the gut microbial population, it implied that the observed reduction of plasma deoxycarnitine in PKU patients could potentially relate to a reduced carnitine biosynthesis in PKU. Because patients receiving free AAs received more carnitine with their supplements than patients receiving GMP-based products, and also showing significantly higher urinary TMAO excretion partially due to carnitine metabolism of gut bacteria, the authors implied that carnitine could result in more bioavailablity when GMP-based products are consumed [198].

To evaluate with more precision how the different gut microbial populations are influenced by the dietary regimen, a study in children with PKU on a low-Phe diet and children with mild hyperphenylalaninaemias on a unrestricted diet compared the microbial biodiversity in faecal samples. Children with PKU displayed a significantly decreased faecal butyrate presence and a significant depletion of *F. prausnitzii* and *Roseburia spp*, two of the most abundant butyrate-producing genera and positively associated with healthy status in patients with hyperphenylalaninaemia [199].

A follow-up of the above-reported study measured different parameters among which a more in-depth evaluation of the gut microbial composition, which was confirmed to be different based on the various dietary regimens of the two study populations. While in both groups Firmicutes and Bacteroidetes were the relatively most abundant phyla, *Veillonellaceae* resulted in being significantly depleted in children with PKU. At the genus level, *Faecalibacterium* and *Ruminococcaceae* (other) were more abundant in subjects with mild hyperphenylalaninaemias, while *Blautia*, *Clostridium* (*Lachnospiraceae* family), and *Lachnospiraceae* (other) were significantly more abundant in children with PKU. Interestingly, correlations with dietary intake showed that the significantly higher presence of *Faecalibacterium* spp. in subjects with mild hyperphenylalaninaemia negatively correlated with both soluble and insoluble fibre intake, as well as with glycaemic index and glycaemic load. Similarly, the *Ruminococcaceae* family, more abundant in subjects with mild hyperphenylalaninaemia, negatively correlated with glycaemic index and soluble fibres, while *Lachnospiraceae* (other) genus correlated positively with these two nutritional parameters. Furthermore, *Oscillospira* was found to positively relate to energy assumption and carbohydrates, while *Roseburia* resulted in being negatively correlated with soluble fibres. Such results, that in part differ from previously reported observations in PKU patients, have been suggested to be related not only to the quantity of consumed carbohydrates, these being quite abundant in the typical low-protein regimen of PKU patients, but also to their quality, affecting, for example, the glycaemic index and insulin resistance. Gene sequencing also revealed that *Ruminococcus bromii*, a starch degrader and a non-butyrate-forming member of the *Ruminococcaceae,* was the indicator species in PKU subjects, indicating a shift towards a less healthy profile in the Firmicutes phylum. *Blautia* spp., known to exert a proinflammatory effect on gut mucosa by inducing cytokine secretion, were also significantly more abundant in patients with PKU than in patients with hyperphenylalaninaemia. These results indicate the need to better investigate gut biodiversity in children and patients with PKU with the objective of modulating the consumption of pre and probiotics adapted to the modified dietary schemes necessary for the management of PKU [200].

Another group analysed the gut microbial biodiversity in patients with PKU and, besides the limitations of the study mainly based on the extremely low number of patients and inadequate technology for complete faecal analyses, showed a significant difference of *E. coli* presence in guts of normal subjects versus children with PKU, where these bacteria were not found. In addition to the obvious need of confirmation, this result is aligned with those measuring differences in the relative abundance of microbial species in the gut of patients with PKU [201].

## 6. Limitations

Detailed information on the role of microbiota in IEMs is scarce, given the rarity of these diseases as well as the presence of subclasses within the same IEM. These few studies normally have a small number of participants, making interpretation complex.

Another aspect to be considered is that the microbiota is mainly influenced by diet, but in IEMs, diet overload or restrictions are part of most common treatments, making the identification of an adequate control group very difficult.

Overall interpretation of data is extremely intricate in a complex environment such as the microbiota, not only influenced by numerous environmental factors, but where the metabolic genetic defect may also affect the microbiome. At this stage is still not possible to discern if a condition observed in patients (i.e., a dysbiotic state) reflects genetic or diet effect.

## 7. Discussion

It is without doubt that the condition of the microbiota for human beings is a key aspect to consider for the wellbeing of healthy subjects and for patients. Unfortunately, its complexity has not allowed clarification of all its roles, effects, and interactions yet. If on one side some “functional” roles of the microbiota are highly conserved, such as the contribution to the production of cofactors and vitamins, the influence on the carbohydrate metabolism, and the aromatic AA and ATP synthesis [202,203], all the environmental factors altering the composition of the microbiota, and primarily the diet [29,204], sustain functional changes that can influence the wellbeing and health of a human being.

In general terms it is known that a dysbiosis, an impairment of the microbiota favouring an abnormal growth of some microbial species over other ones or a reduced microbial diversity resulting in diminished abundance of some important species, has the potential to affect the health of human subjects. The presence and the quantity of microbial species in the human gut interact with the body because bacteria metabolize food and drugs and because metabolites are released by microbial metabolism. These metabolites are then distributed via blood circulation to the different body organs with the potential to exert positive or negative effects. To be able to proactively modulate the microbiota it is mandatory to continue analysing data and gaining precise understanding, for example assessing the metabolome. Some indicators of how microbiota may be predictive of human diseases have already been defined. For example, elevated TMAO levels seem to be predictive of cardiovascular problems in subjects with cardiovascular history [205,206,207] or metabolites of Trp catabolism (e.g., indolepropionic acid) can be associated with the aetiology of different diseases based on the observation that different sets of Trp metabolizing bacterial pathways coexist with either endocrine, inflammatory processes particularly active in the gut:brain axis [208], or both. Albeit they are good indicators, they are far from being precise and do not take into account interfering factors (such as effects of medications interfering with microbiota or gender and race differences) and more research is highly demanded.

In patients with IEMs the balance of the microbiota may be at higher risk due to the underlying disease and the very frequent need or obligation to observe a special dietary regimen, devoid of those elements or nutrients that the patient is not able to metabolize to reduce the risk of accumulating toxic compounds. In fact, patients with IEMs have a higher risk than the healthy population of modifying their metabolism and physiology with the ultimate result of affecting some pathways and processes in their bodies [209].

Among the various metabolic and functional changes that a modified microbiota may sustain, the implications on the gut-brain axis are particularly relevant when subjects are obliged to observe restricted/modified diets over many years or life-long. Being a two-way communication pattern, stressful situations affecting the brain may affect the gut microbiome via the hypothalamic-pituitary-adrenal axis, with consequences on the immune activity or bowel function [210], while an unbalanced microbiota composition can sustain the secretion of proinflammatory cytokines, in turn associated with neuropsychiatric disorders such as depression, anxiety, and others [211,212]. Of the numerous consequences an unbalanced gut microbiota may have on wellbeing and behaviours in light of the gut-brain axis connections, it may be particularly interesting to underline the influence of microbiota on anxiety and depression. It is known that some patients with PKU, despite a good lifelong dietary control, have an elevated risk of mood, anxiety, and attentional disorders [213]. So far, the recommendation is to uninterruptedly follow the dietary regimen to control the disease, although this is not the only factor potentially triggering anxiety, depression, and cognitive impairment. For example, alterations of gut microbiota composition have been observed in subjects simultaneously presenting anxiety and gastrointestinal barrier dysfunction [151]. Sustained by extensive research in murine models, the association between microbiota composition and anxiety and mood have also been observed in the general population receiving a mixture of *Lactobacillus helveticus* R0052 and *Bifidobacterium longum* R0175 for 30 days where anxiety and stress response were reduced and mood was improved [214]. Similarly, a triple-blind, placebo-controlled, randomized study with 20 healthy subjects without mood disorder receiving probiotics (*Bifidobacterium bifidum*, *Bifidobacterium lactis*, *Lactobacillus acidophilus*, *Lactobacillus brevis*, *Lactobacillus casei*, *Lactobacillus salivarius*, and *Lactococcus lactis*) for 4 weeks showed that negative thoughts associated with sad mood were reduced [173].

Another factor not to forget is the opportunity or the necessity, in some of the IEMs, to also treat patients pharmacologically. For example, in propionic and methylmalonic acidaemias metronidazole has been used for many years with the intent of reducing the intestinal bacteria responsible for the production of propionate [179]. Research has afterwards demonstrated that this antibiotic was not efficacious in reducing SCFA production [215], while long-term use of such antibiotic could promote development of antibiotic resistance or cause toxicity at the central nervous system level [216]. Reports of use of metronidazole in urea cycle disorders to reduce the overall ammonia load exist, but lacking data confirming the utility of such use this antibiotic is not recommended to obtain the desired reduction of nitrogen amounts [217] highlighting the need of careful evaluation of the complete treatment approach to the disease. Indeed, in the latest guidelines for the diagnosis and management of urea cycle disorders the use of metronidazole is not considered for long-term treatment [217].

Specific knowledge of the effects of microbiota both on system-wide or organ-specific metabolism will be more and more helpful to therapeutically approach IEMs. Measurement of faecal samples represents an easy technique to start evaluating how microbiota are able to influence certain pathways of metabolism, primarily that of SCFAs, heterocyclic amine, and bile acids [218,219,220].

The implementation of analytical approaches, such as metabolomics able to reveal more comprehensive metabolic signatures, it will be possible to intervene with precision on the therapeutic approach of IEMs, taking into account, among others, the role of diet and the microbial influences on host metabolism.

As much as these initial results warrant further research and demonstrations, there may be opportunities to intervene with probiotics to modulate the wellbeing of patients with IEMs, and PKU in particular. As PKU is one of the most studied IEMs and still finds in the dietary approach a consistent, if not unique, portion of the therapeutic treatment, the angle of the microbiota composition and modulation should be taken in high consideration in the holistic approach to this disorder. In these patients the modified diet followed to minimize the consumption of Phe could create unbalances in the ratio of Firmicutes /Bacteriodetes, a factor that has been associated with an increase of BMI in human subjects and, dangerously, to childhood obesity. Coupled with chronic reduced consumption of AAs, often not compensated enough by protein substitutes, the microbiota of patients with PKU may result in importantly modified, shifting towards a microbial composition potentially favouring a less efficient glycaemic control, increased insulin resistance, and weight gain.

The few studies that analysed the composition of the microbiota in PKU patients confirm the observation that a chronically modified diet influences the microbiota composition. In very young children (mean age 4.2 years of age) with PKU, de Oliveira F. et al. [194] showed that among the most represented phyla, Firmicutes were less prevalent when compared with the corresponding concentration in healthy subjects. Furthermore, they showed a less diverse and different microbial population in the intestines of PKU patients. The latter presented a reduced concentration of *Clostridiaceae*, *Erysipelotrichaceae* and *Lachnospiraceae* families, *Clostridiales* class, *Coprococcus*, *Dorea*, *Lachnospira*, *Odoribacter*, *Ruminococcus* and *Veillonella* genera than in control subjects. On the contrary, bacteria of the genera *Akkermansia* and *Prevotella* and the Peptostreptococcaceae family were more represented in the PKU group. Interestingly, the Phylogenetic Investigation of Communities by Reconstruction of Unobserved States analysis done on the samples, indicated which could be the functional prediction of such a significantly modified microbiota, as measured in the PKU subjects. In fact, up to 23 bacterial functions resulted in being underrepresented in the PKU group, with potential consequences on starch and sucrose metabolism, glycolysis/gluconeogenesis, as well as Phe, Tyr, Trp, valine, leucine, and isoleucine biosynthesis. Of the overrepresented species in PKU subjects, 26 were related to lipopolysaccharide biosynthesis proteins and to glutathione metabolism. The authors imply that one of the strongest factors at the basis of these modifications is the higher levels of Phe in the blood of PKU subjects vs. the controls, although many other environmental factors need to be taken into account to justify the observed differences. Of interest is certainly the observation that the functional prediction model seems to corroborate the connection between higher carbohydrate and lower lipid intake of the PKU young children with the microbial composition, in turn associated with modification of starch and glucose metabolism. As an impaired energy harvesting capacity could potentially lead to overweight and obesity, such factors should already be taken into account in PKU subjects experiencing an unbalanced diet since the first years of their lives.

In children of a median of 8.7 years old, Verduci E. et al. [199] evaluated the composition of the microbiota comparing it to a matched population of subjects with untreated hyperphenylalaninaemia (MHP; blood Phe 120–360 mmol/L). Despite the different control groups, the different median age of the sample and the different analysis methods of the de Oliveira F. et al. study [194], in this study the composition of the microbiota of PKU subjects was also less diverse when compared to the controls. Furthermore, a significant reduction of total SCFAs and butyrate production was observed in PKU patients, correlating with a significantly reduced depletion of the butyrate-producing *F. prausnitzii* and *Roseburia* spp. and of the lactate-producing *Lactobacillus* spp. As the different diets followed by patients with PKU and MHP subjects do not seem to support the observed differences in SCFA concentrations, the authors suggest the microbiota, related to the reduced abundance of *F. prausnitzii* and *Roseburia* spp., as primarily involved in this variation. In addition, the measurement of the glycaemic index and load showing significantly increased values in PKU patients could be the basis of the modified gut microbial composition by providing a different substrate, although this cannot be considered the only cause, as the disease itself could be a direct cause for the observed differences in the microbial population. In larger patient PKU and MHP populations, Bassanini G. et al. [200] corroborated the observation of the Verduci E. et al. [194] study that the relative abundance of Firmicutes and Bacteroidetes was different, with the Firmicutes phylum slightly less abundant in PKU subjects, but with *Veillonellaceae* significantly depleted in these patients. Furthermore, *Faecalibacterium* and *Ruminococcaceae* (other) were significantly more abundant in MHP children, while *Blautia*, *Clostridium* (of the *Lachnospiraceae* family), *Lachnospiraceae* (other), and other species of the Firmicutes phylum were significantly increased in the PKU subjects when compared with the MHP controls. These changes once again indicate that the changes in the species’ presence as well as their relative abundance were modified in PKU patients with respect to subjects following a regular diet. Consequences can influence the host metabolism, like for example, facing a chronic lower SCFA content, especially of butyrate, due to the relatively reduced abundance of the butyrate-producing species, while hosting more samples of other genera (i.e., *Blautia*) that have been associated with proinflammatory effects on the gut mucosa. Another aspect to take into consideration is the response of the Firmicutes phylum when evaluating daily glycaemic index and glycaemic load. The negative correlation between *F. prausnitzii* with the two indexes could be related especially to the quality of the carbohydrates. Fast-absorbed carbohydrates retain a high glycaemic index and seem to be correlated with a reduction of *F. prausnitzii* concentration in subjects at risk of metabolic syndrome, making an informed and careful composition of the low-protein foods an aspect potentially ameliorating the quality of foods available for this patient population.

The interest in the effects of food has encouraged research to shed light on the role of pre and probiotics for patients with IEMs, particularly PKU. A few strategies have been assessed over the years in order to modulate the intestinal microbiota in patients with PKU, starting with the addition of prebiotic oligosaccharides in infant formula that helped maintain a predominant concentration of bifidobacteria and reduce the pH of stools, two factors associated with reduced risk of infection in healthy breast-fed infants and therefore potentially favourable for PKU patients [193].

In this field, the research on the features of the GMP polypeptide has spanned in different directions to find that GMP can bind and inactivate toxins of *E. coli* and *Vibrio cholerae* [221], inhibit the adhesion of cariogenic bacteria [222], enhance zinc absorption [223], increase cytokine production [224] and decrease stimulation of T-helper1 lymphocytes in rats [225], and act as an anti-inflammatory in different models [226,227]. Among others, GMP has been studied for the control of satiety, although with conflicting results [228,229,230], for the effects on bone size and strength [231,232,233], and interestingly for its role as prebiotic. The latter is thought to be based on the fact that GMP shows a high degree of glycosylation that includes mucin-like oligosaccharide chains that contain sialic acid, galactose, and galactosamine. In healthy and PKU rats, GMP has been found able to modulate the microbiota by reducing the presence of *Desulfovibrio*, a sulfate-metabolizing bacterium, in favour of increases in the Bacteroidetes or Firmicutes phyla, and was also associated with increased levels of SCFAs as well as anti-inflammatory effects [186]. Interestingly, the addition of GMP in the medium containing human faecal samples supports diversity of microbiota and higher total SCFAs. The increased abundance of *Coprococcus* and *Dorea,* if compared with samples whose medium was not enriched with GMP, seems to be a favourable signal, given the association of the presence of these species to resistance to pathobiont colonisation [189]. As much as all these positive indicators need confirmation in large scale human studies, GMP has been developed to be used in medical foods for PKU patients, despite a very low residual Phe content due to processing, and as such has been evaluated in patients for numerous aspects that are thought to be advantageous when patients consume GMP-based products regularly. Its prebiotic features, also observed as promoting bioavailability of Tyr [197], could contribute to provide patients with PKU with a product favouring their wellbeing in many ways.

**Figure 3:**
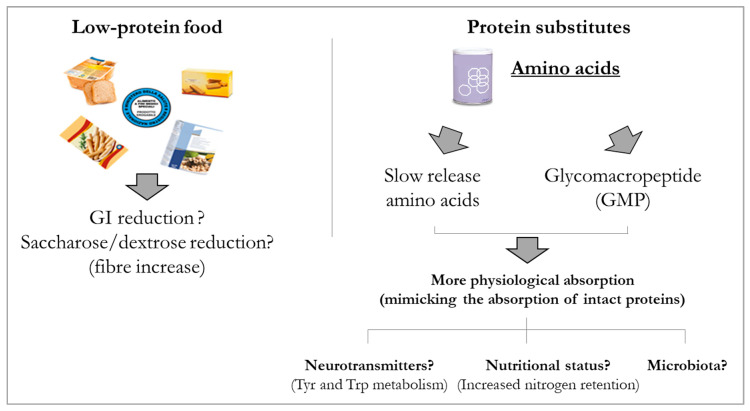
Modulation of dietary approach in PKU. The provision of low-protein foods that are balanced in their content and the quality of carbohydrates may favour patients with PKU in better controlling the composition of the microbiota and as a consequence the host metabolism connected to microbial metabolic processes. Phe-free AAs are the mainstay of the dietary approach to PKU and their integration in a dietary regimen poor of natural proteins needed to reduce the intake of Phe is key to patient wellbeing. New options with AAs deriving from natural sources or specifically engineered to provide absorption features closer to intact proteins are available for patients with PKU. A different way of administering AAs could prove favourable for patient with PKU by modulating some of the imbalances typical of the PKU diet.

In general, the approach to the dietary regimen for patients with PKU can be enriched with different considerations, in addition to the obvious control of Phe intake, to positively affect the way AAs are absorbed, the consequences of the diet composition and AA kinetics on the gut:brain axis pathways and the relative composition of the microbiota (Figure 3).

In addition to these approaches possible with specific modifications in the composition or production process of the products used in PKU, other approaches have been taken in order to further improve the control of the disorder and the wellbeing of patients. 

Aside from the pharmacological approaches, there is a wealth of research aiming at using specific species of bacteria as pre or probiotics, or utilizing the bacteria as vectors of Phe hydroxylase (PAH) or Phe lyase (PAL) to be able to give the patient the possibility to metabolize Phe and reduce or eliminate dependence on the life-long low-protein diet. As a probiotic, *E. coli* Nissle has been extensively studied in animal models for its positive features and persistence in conditions of low microbial diversity, a factor linked to a variety of pathological or disease-related situations, also making it a good candidate as a vector for Phe-metabolizing PAL genome [192]. *Lactobacillus reuteri* [190] and again *E. coli* Nissle [191] have been used to express PAH with the aim of metabolizing Phe before it is absorbed, using animal models and following tests in human subjects, proving that this is a possibility. It takes more time before products of this kind are available for use in patient with PKU but they pave the way for coupling synthetic biology and microbiome research to become a viable therapeutic modality.

## 8. Conclusions

In conclusion, the role of microbiota is continuously under scrutiny to better understand how its modulation may support educated approaches to improvement of the wellbeing of patients with IEMs. A holistic approach to the disorder looks at the variety of factors influencing the quality of life, and ultimately the health, of patients, and could strongly benefit from a personalized approach based on the knowledge about how components of the diet or concomitant drugs may modulate the microbiota and how the microbiota may in turn influence host metabolisms and neurological functions. The modified and reduced microbial diversity has been associated to a reduction in the production of beneficial SCFAs and butyrate and an increased release of pro-inflammatory molecules. In order to reduce imbalances, viable approaches, as in the healthy population, patients with IEMs, and particularly PKU, may use an improvement of quality of diet. Further clinical studies on pre, pro, and postbiotics in IEMs to create a microbial environment that facilitates the management of the disorder and improves quality of life are required. Other approaches, mainly pharmacological and genetic engineering, add additional weapons to the treatment of IEMs that could, in the future, reduce the differences between patients with IEMs and healthy subjects.

## Figures and Tables

**Table 1:** Association between diet and potential microbiota alterations.

Nutrients		Function	Gut Microbiota Alteration
Carbohydrates	Starches, fibres, and glycogen [58,59,60,61]	Short chain fatty acid production via microbiota fermentation	Increase in *Bifidobacterium* spp., Bacteroidetes, *Akkermansia muciniphila*, *Clostridium* spp., and *Prevotella* spp.
	Simple sugars or sweeteners [14]	Enhance the risk of glucose intolerance	Increase of *Bacteroides* genus and Clostridiales order
Proteins	Proteins and amino acids [62,63,64,65]	Enhance the intestinal barrier function and exert an important role in immune and anti oxidative responses	Decrease in *Bifidobacterium* spp. and increase the ratio Bacteroidetes/Firmicutes
Fats	Saturated [66,67,68]	Increased intestinal permeability	Increased ratio of gram-negative intestinal species
	Unsaturated [66,67,68]	Indirect interaction with the gut microbiota	Increase in *Lactobacillus* spp., and *Akkermansia muciniphila*
